# Supplementary material for: Genetic and chemical inhibition of IRF5 suppresses pre-existing mouse lupus-like disease
Source: Nat Commun. 2021 Jul 19;12:4379. doi: 10.1038/s41467-021-24609-4 (PMC8290003; doi:10.1038/s41467-021-24609-4)
Supplement: Supplementary file 1 — Supplementary Information [file 41467_2021_24609_MOESM1_ESM.pdf]

Supplementary Information

Genetic and chemical inhibition of IRF5 suppresses pre-existing mouse lupus-like disease

Tatsuma Ban, Masako Kikuchi, Go R. Sato, Akio Manabe, Noriko Tagata, Kayo Harita, Akira Nishiyama, Kenichi Nishimura, Ryusuke Yoshimi, Yohei Kirino, Hideyuki Yanai, Yoshiko Matsumoto, Shuichi Suzuki, Hiroe Hihara, Masashi Ito, Kappei Tsukahara, Kentaro Yoshimatsu, Tadashi Yamamoto, Tadatsugu Taniguchi, Hideaki Nakajima, Shuichi Ito, and Tomohiko Tamura

Supplementary Figure 1

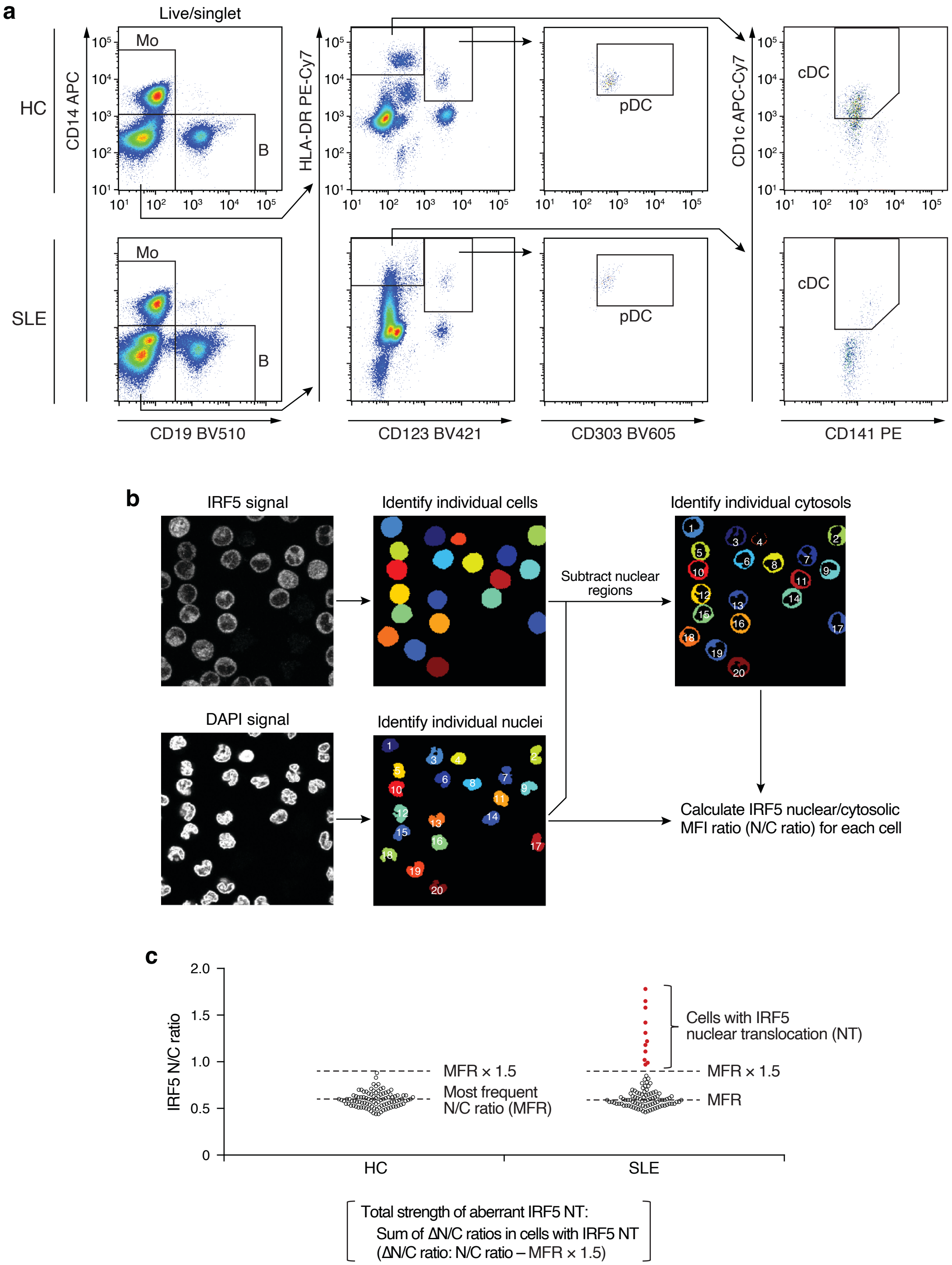

Supplementary Figure 1. Evaluation of IRF5 activation by nuclear translocation.
a, The gating strategy for sorting of monocytes (Mo), B cells (abbreviated as B), pDCs, and cDCs from PBMCs of healthy control (HC) donors or SLE patients. **b**, The flowchart of image analysis. Fluorescent images of sorted cells captured by the confocal microscope were analyzed by CellProfiler as indicated. **c**, The scheme of IRF5 nuclear translocation (NT) evaluation.

Supplementary Figure 2

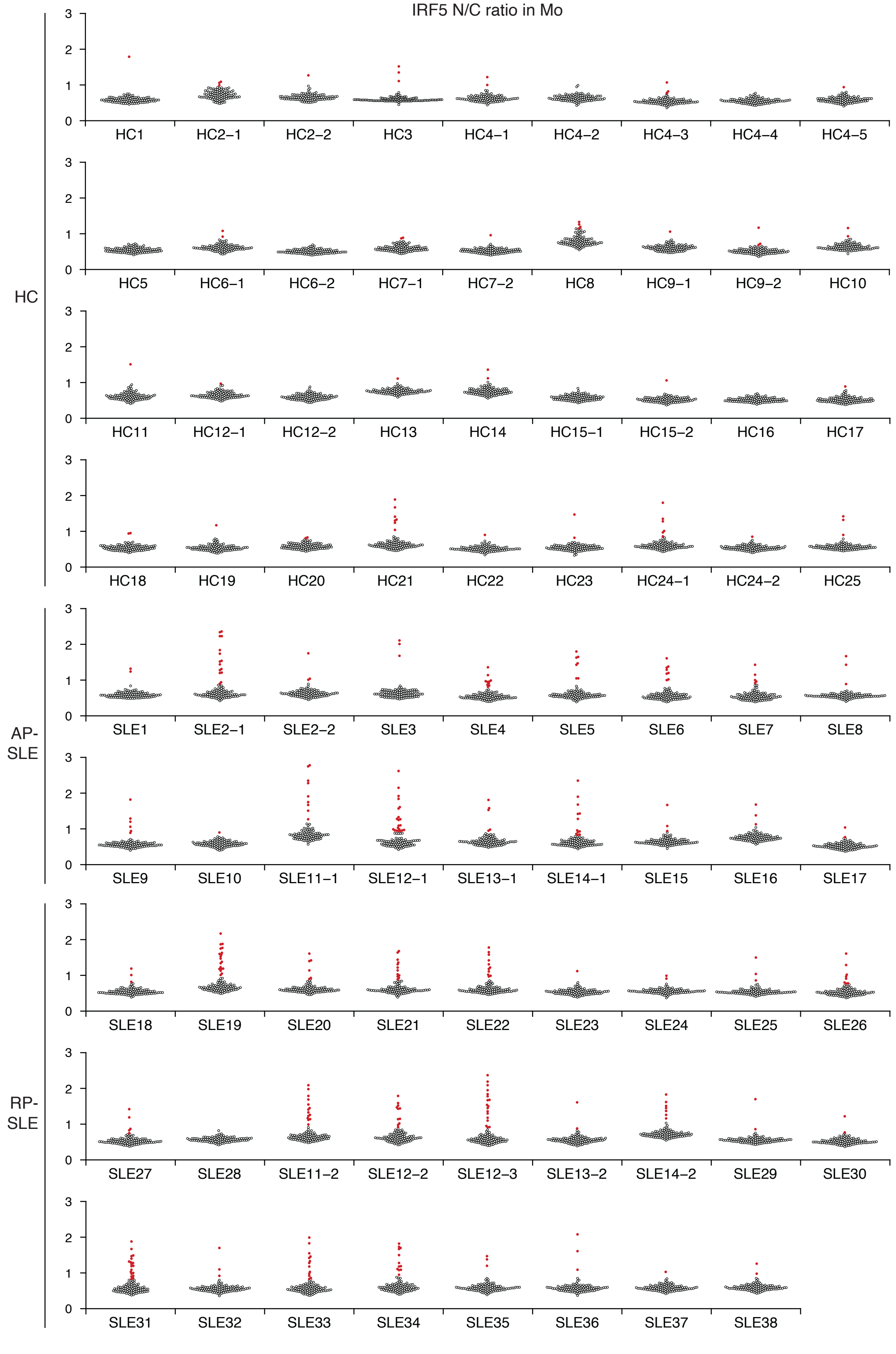

Supplementary Figure 2. Quantitative analysis of IRF5 nuclear translocation.
Data on monocytes from 36 HC donor samples (25 individuals) and 44 SLE patient samples are shown (38 individuals; multiple samples were collected from some patients during the disease course).

Supplementary Figure 3

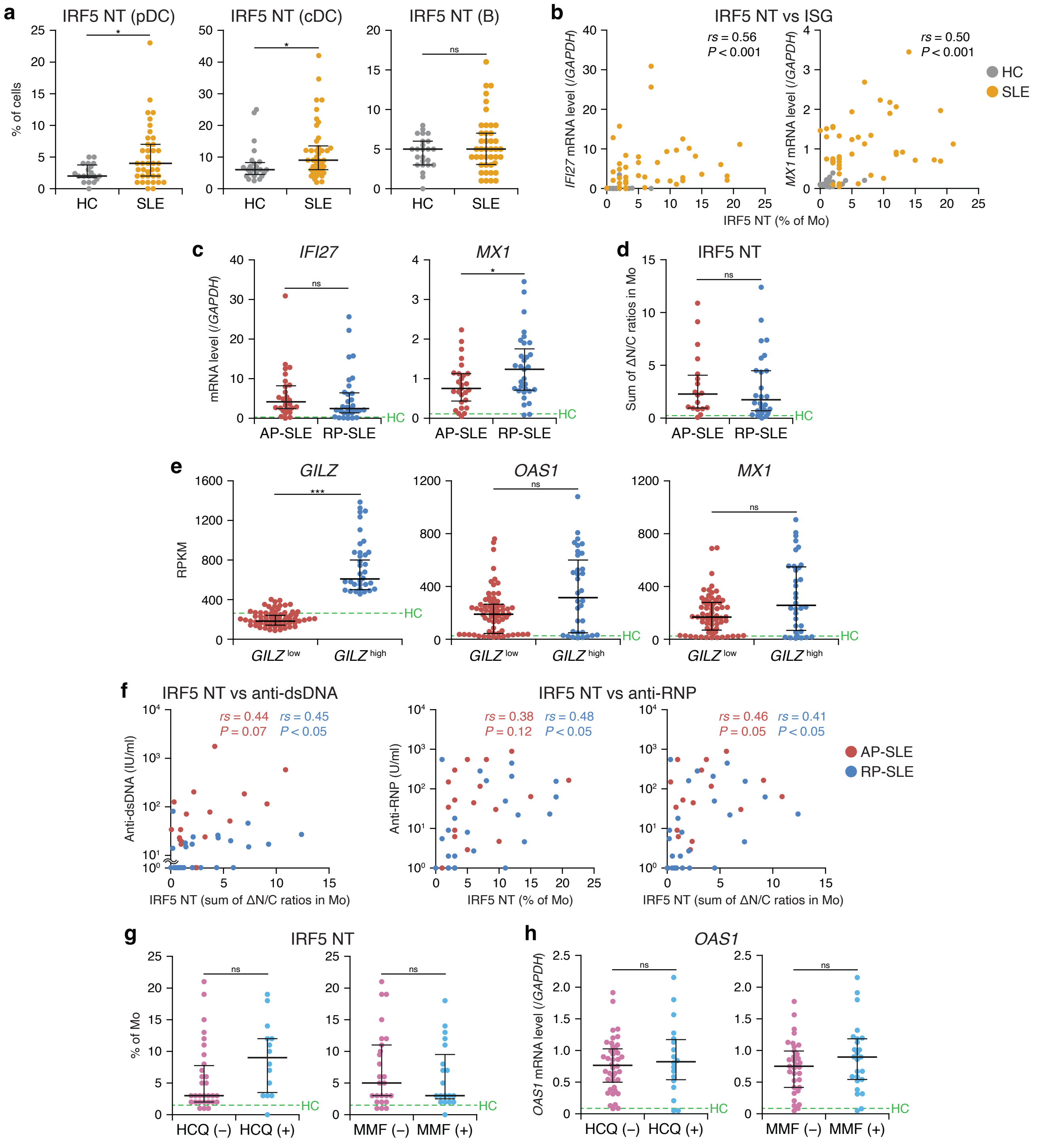

Supplementary Figure 3. Analysis of IRF5 activation status in human SLE.
a, IRF5 NT in pDCs, cDCs, and B cells from HC donors (n = 19, 23, and 24, respectively) or SLE patients (n = 39, 40, and 42, respectively). Data from identical individuals are averaged (HC) or presented separately (SLE patients) here or below. **b**, Correlation between IRF5 NT and ISG expression. Scatterplots of IRF5 NT prevalence among monocytes as well as peripheral-blood *IFI27* (left) or *MX1* (right) mRNA levels are depicted. HC: n = 25, SLE: n = 44. **c**, ISG expression. *IFI27* and MX1 mRNA levels of peripheral blood in AP- and RP-SLE were analyzed by RT-qPCR. **d**, IRF5 activation. The strength of aberrant IRF5 NT in AP- and RP-SLE were analyzed. **e**, Analysis of public RNA-seq data from SLE patients and HC donors. *GILZ*, *IFI27*, and *MX1* expression (RPKM: reads per kilobase of transcript per million mapped reads) of peripheral blood is shown. SLE patients were subdivided into two groups, *GILZ* ^{low} (n = 65) and *GILZ* ^{high} (n = 34), with the threshold of the mean + 1.96 × SD of the *GILZ* mRNA level in HC. **f**, Correlation between IRF5 activation and autoantibody production. Scatterplots of the IRF5 NT in monocytes and serum concentration of anti-dsDNA (left) and anti-ribonucleoprotein (RNP; middle and right) in AP- and RP-SLE are provided. **g**,**h**, Aberrant IRF5 activation and ISG expression. IRF5 NT in monocytes (**g**) or the *OAS1* mRNA level (**h**) in SLE patients treated (+) or not treated (−) with either HCQ (left panel) or MMF (right panel). AP-SLE: n = 27 (**c**) or 18 (**d**,**f**), RP-SLE: n = 31 (**c**) or 26 (**d**,**f**), HCQ (−): n = 30 (**g**) or 40 (**h**), HCQ (+): n = 14 (**g**) or 18 (**h**), MMF (−): n = 25 (**g**) or 33 (**h**), MMF (+): n = 19 (**g**) or 25 (**h**). Horizontal bars denote median with interquartile range (**a**,**c**–**e**,**g**,**h**). Dashed lines (**c**–**e**,**g**,**h**) indicate the median of HC data. *$P < 0.05$, ***$P < 0.001$ (two-sided Mann-Whitney *U* test in **a**,**c**–**e**,**g**,**h**). Spearman's rank correlation coefficient (*rs*) and *P* value were utilized to assess the correlation (**b**,**f**).

Supplementary Figure 4

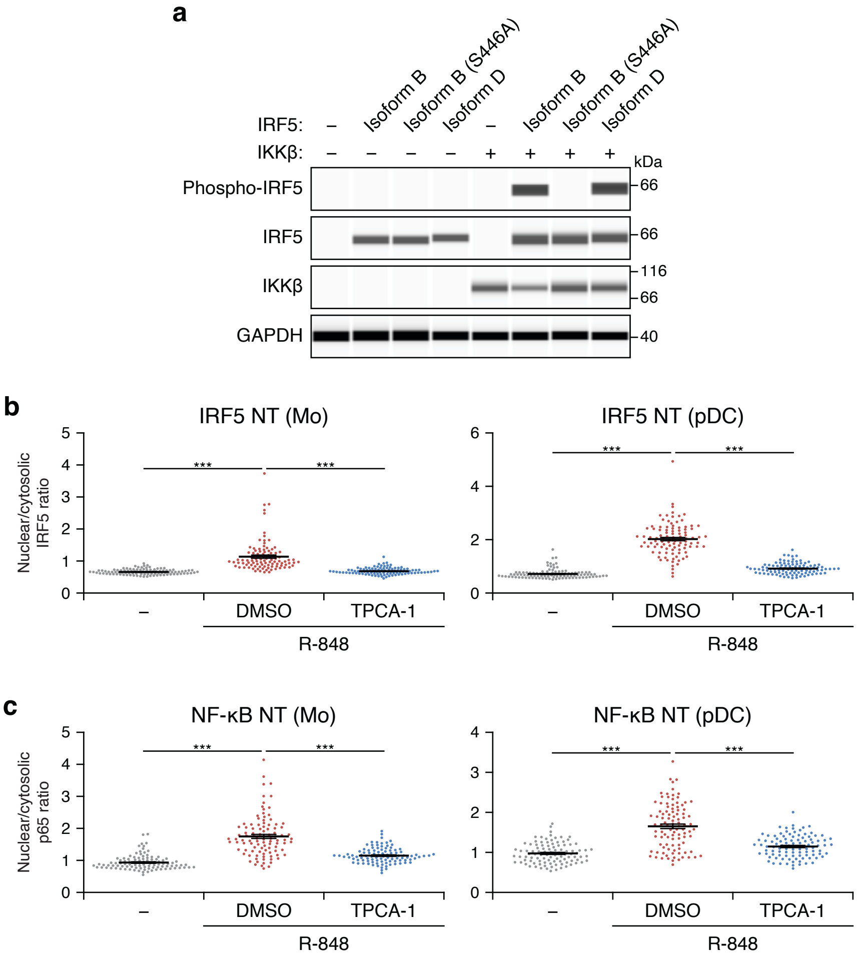

Supplementary Figure 4. Analysis of IRF5 activation in human cells.

a, Specificity of the anti-phospho IRF5 monoclonal antibody. HEK293T cells were co-transfected with human IRF5 (WT isoform B, a mutant isoform B with alanine substitution of the serine 446 [S446A], or WT isoform D) and/or IKKβ. The cell lysates were analyzed via a capillary-based immunoassay using antibodies against phosphorylated IRF5 (phospho-IRF5), total IRF5, IKKβ, and GAPDH as a loading control. **b**,**c**, The influence of the IKKβ inhibitor on NT of IRF5 and NF-κB. The nuclear/cytosolic ratio of IRF5 (**b**) or NF-κB p65 (**c**) in 100 monocytes (left panel) or pDCs (right panel) sorted from human HC PBMCs pretreated with either DMSO or 1 μM TPCA-1 for 30 min and then stimulated with 3 μM R-848 for 120 min. Data are representative of two independent experiments. Horizontal bars denote mean ± SEM (**b**,**c**). ***$P < 0.001$ (two-sided Student's *t*-test in **b**,**c**).

Supplementary Figure 5

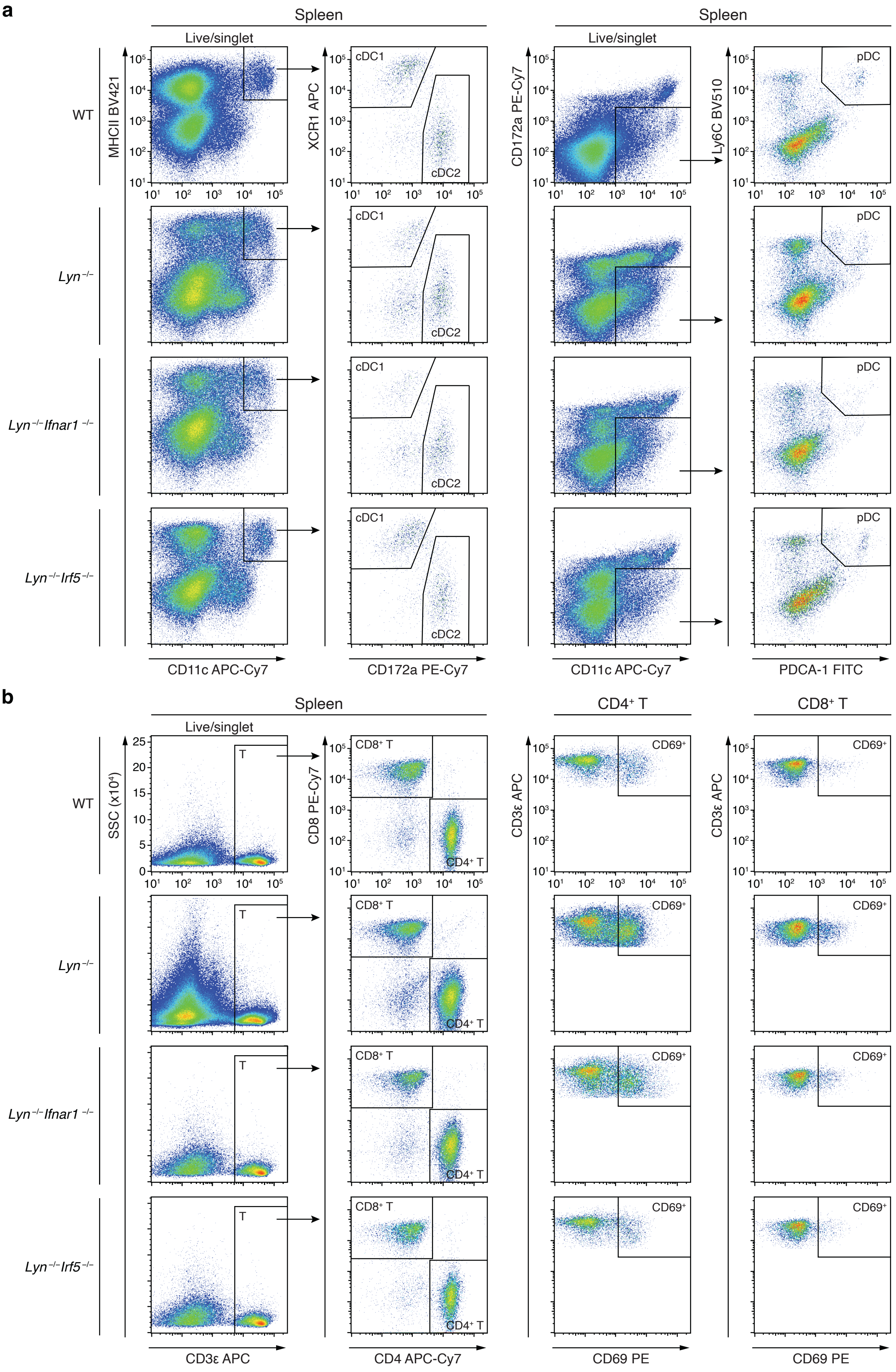

Supplementary Figure 5. Flow cytometric analysis of mouse immune cell subsets.
a,**b**, Gating strategy for the flow cytometric analysis of splenic DC subsets (**a**) and T cells (**b**).

Supplementary Figure 6

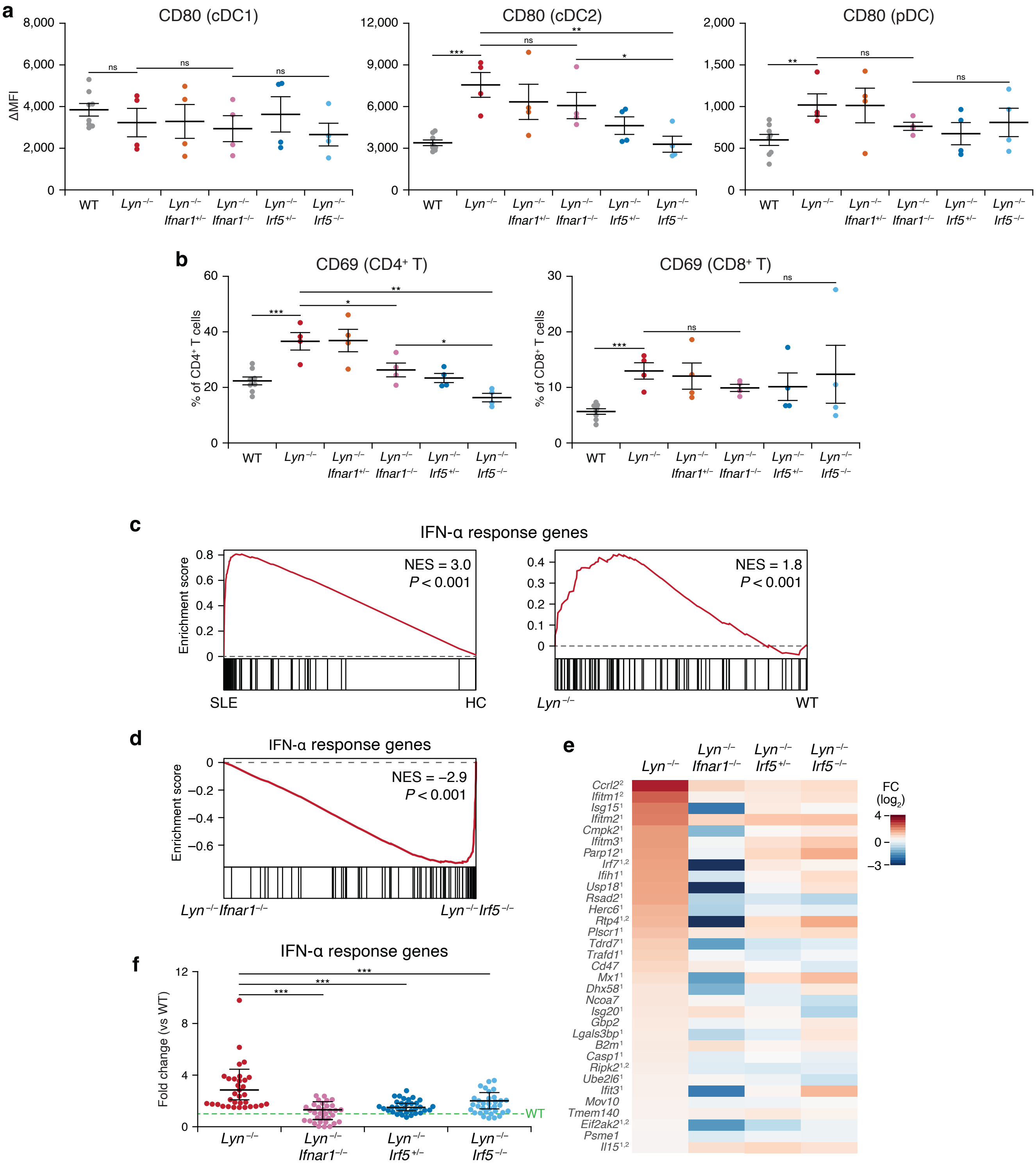

Supplementary Figure 6. Innate and adaptive immune cell activation and IFN-α response genes in the mouse SLE model lacking *Ifnar1* or *Irf5*.

a,**b**, Activation of DCs and T cells. CD80 expression level (ΔMFI; MFI for CD80 minus that of the control IgG) in DCs (**a**) and frequency of $CD69^+$ cells in $CD4^+$ and $CD8^+$ T cells (**b**) from WT, $Lyn^{-/-}$, $Lyn^{-/-}Ifnar1^{+/-}$, $Lyn^{-/-}Ifnar1^{-/-}$, $Lyn^{-/-}Irf5^{+/-}$, and $Lyn^{-/-}Irf5^{-/-}$ female mice at 33–39 weeks of age (n = 8 and n = 4 for WT and each of other genotypes, respectively). **c**,**d**, A GSEA enrichment plot of IFN-α response genes (human SLE versus [vs] HC or $Lyn^{-/-}$ vs WT in **c**, $Lyn^{-/-}Ifnar1^{-/-}$ vs $Lyn^{-/-}Irf5^{-/-}$ in **d**). **e**, A heatmap of the expression of IFN-α response genes in indicated genotypes. The color represents the mean fold change (FC) relative to WT. Genes with FC > 1.5 in $Lyn^{-/-}$ mice are shown. The superscript number on the gene name denotes the gene included in the gene set of the IFN-γ response ([1]) or inflammatory response ([2]). **f**, A dot plot of the data from **e** (n = 33 for each genotype). The horizontal bars represent mean ± SEM (**a**,**b**) or median with interquartile range (**f**). Data in **a**,**b** were compiled from four independent experiments. $^*P < 0.05$, $^{**}P < 0.01$, $^{***}P < 0.001$, ns: not significant (two-sided Student's *t*-test in **a**,**b**; two-sided Mann-Whitney *U* test in **f**).

Supplementary Figure 7

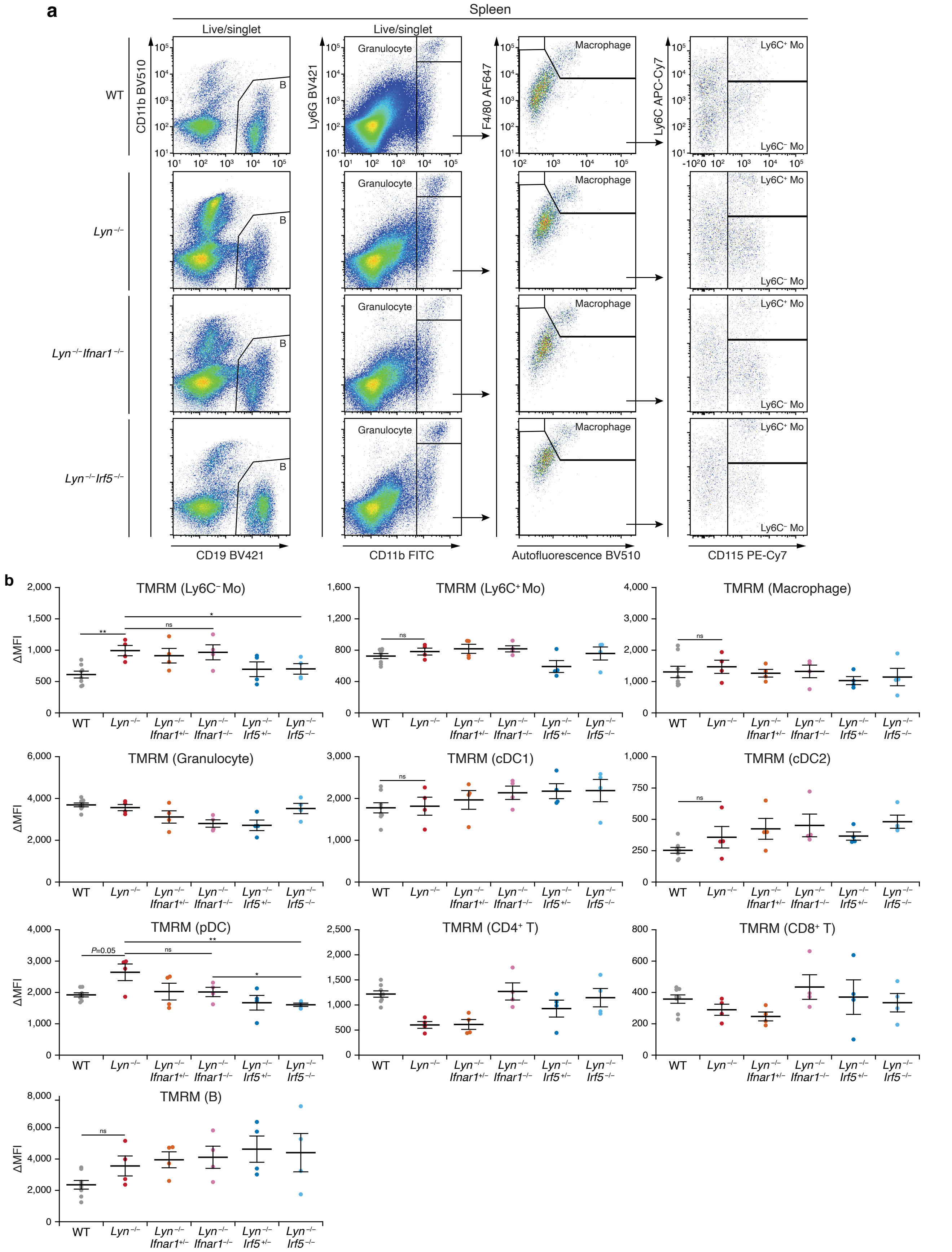

Supplementary Figure 7. Mitochondrial membrane potential in the mouse SLE model lacking *Ifnar1* or *Irf5*.
a, Gating strategy for splenic B cells, granulocytes, macrophages, and Ly6C⁻ and Ly6C⁺ monocytes (Mo). The gating strategies for DC subsets and T cells were similar to those for Supplementary Fig. 5a and b, respectively. **b**, TMRM signal (ΔMFI; MFI for TMRM minus that of unstained control) in the indicated cell types from mice in Supplementary Fig. 6a,b. The horizontal bars represent mean ± SEM (**b**). Data in **b** were compiled from four independent experiments. $*P < 0.05$, $**P < 0.01$, ns: not significant (two-sided Student's *t*-test in **b**).

Supplementary Figure 8

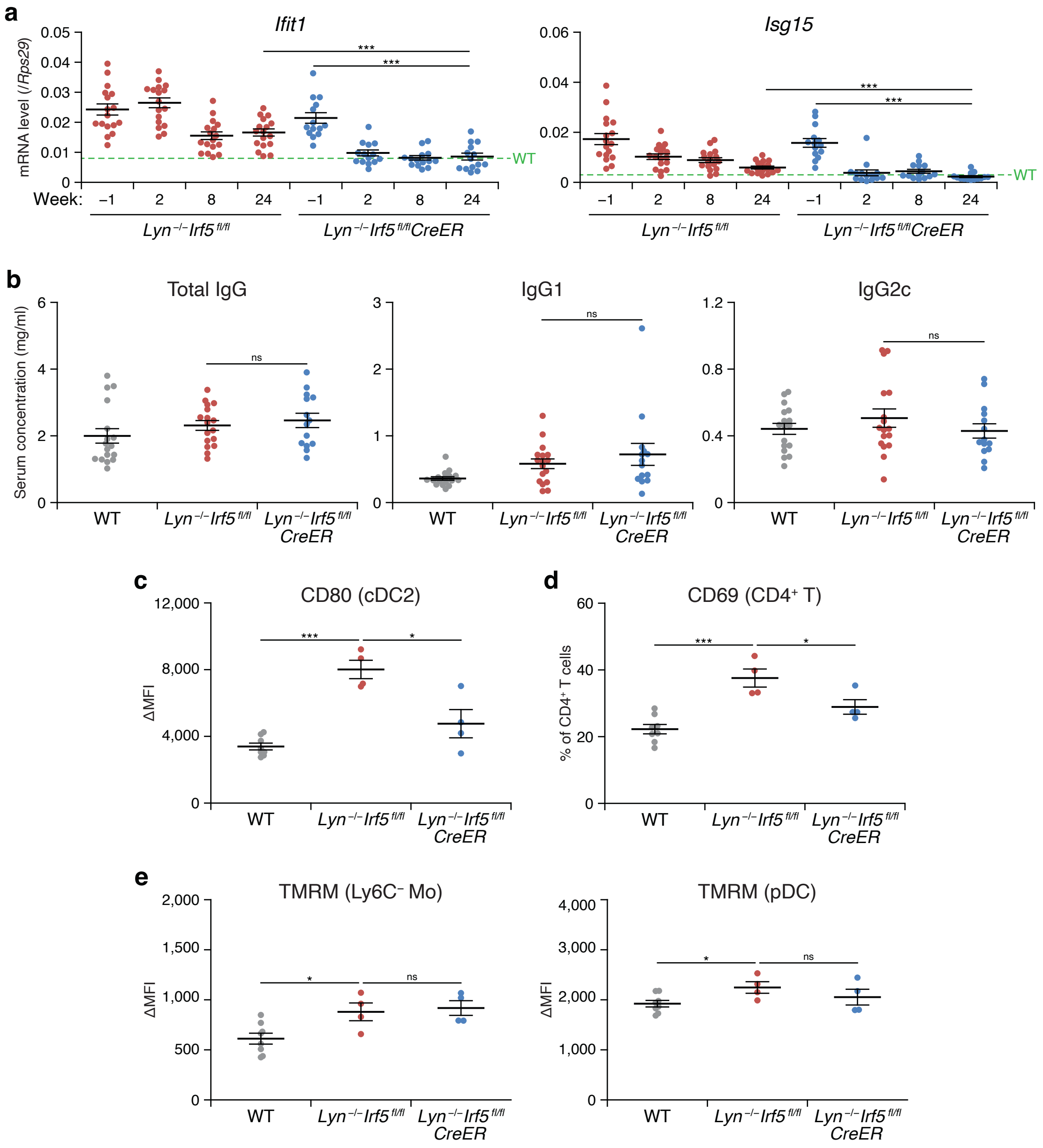

Supplementary Figure 8. ISG expression and IgG production, innate and adaptive immune cell activation, and mitochondrial membrane potential in *Irf5* cKO mice.
a, *Ifit1* and *Isg15* mRNA levels in peripheral blood from the mice in Fig. 3b at the indicated time points (n = 17 [*Lyn*$^{-/-}$*Irf5*$^{fl/fl}$] and 14 [*Lyn*$^{-/-}$*Irf5*$^{fl/fl}$*CreER*] per time point) were analyzed by RT-qPCR. Dashed lines denote the mean of WT mouse data (n = 17). **b**, Concentration of serum total IgG, IgG1, and IgG2c from the mice in Fig. 3b at 24 weeks after the TAM treatment. **c**–**e**, CD80 expression level in cDC2s, frequency of CD69$^+$CD4$^+$ T cells, and TMRM signal in WT (n = 8), *Lyn*$^{-/-}$*Irf5*$^{fl/fl}$ (n = 4), and *Lyn*$^{-/-}$*Irf5*$^{fl/fl}$*CreER* (n = 4) female mice at 4–7 weeks after TAM treatment (36–39 weeks of age). Data in **c**–**e** were compiled from four independent experiments. Horizontal bars denote mean ± SEM. *$P < 0.05$, ***$P < 0.001$, ns: not significant (two-sided Student's *t*-test for comparison between genotypes in **b**–**e** and two-sided paired-*t* test for comparison within the same genotype in **a**).

Supplementary Figure 9

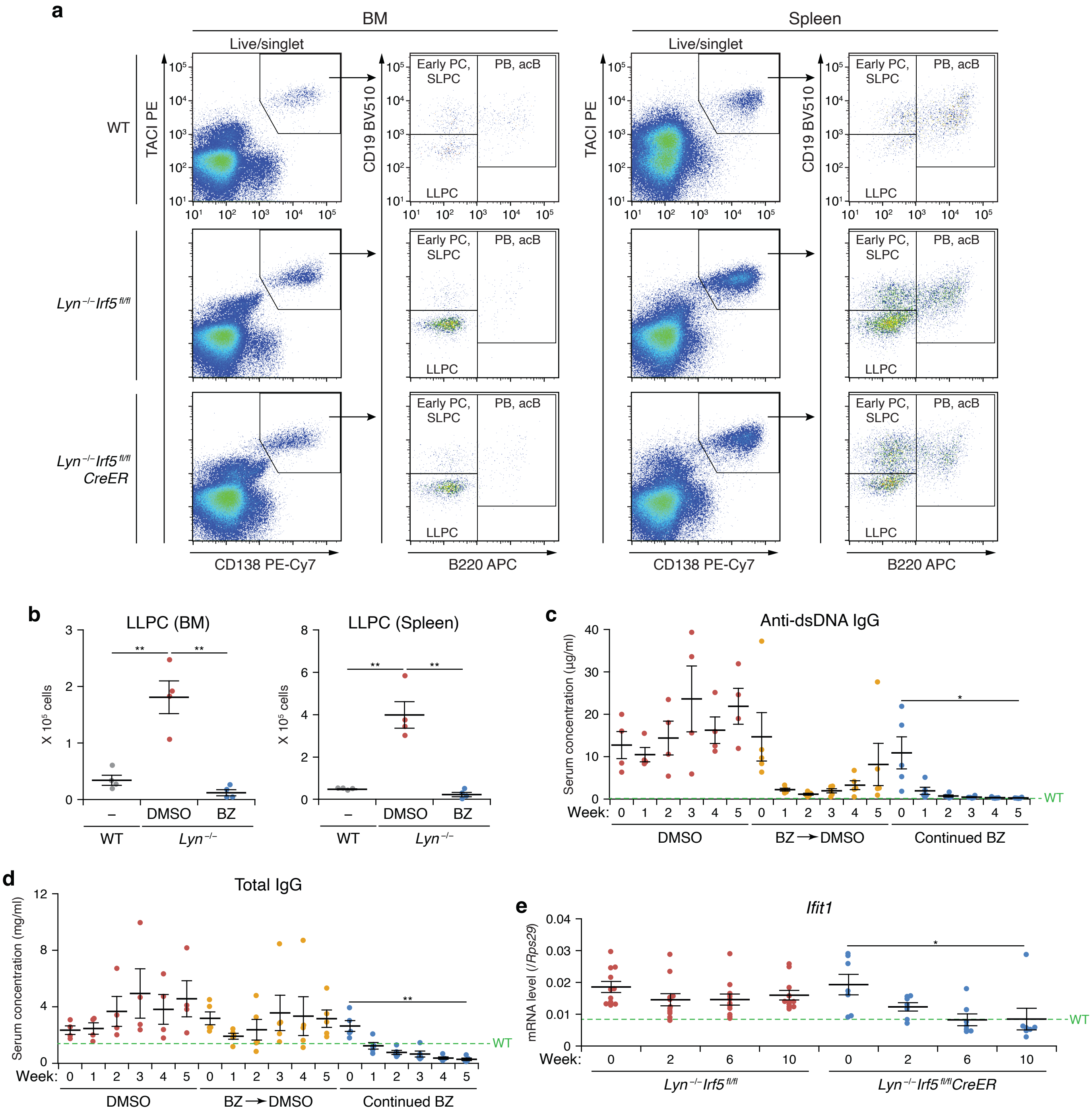

Supplementary Figure 9. The influence of BZ treatment on the Lyn-deficient mouse model of SLE.

a, The gating strategy for flow cytometric analysis of LLPCs. BM: bone marrow, PB: plasma blasts, PC: plasma cells, SLPC: short-lived plasma cells, and acB: activated B cells. **b**, LLPC depletion under the influence of BZ. *Lyn*$^{-/-}$ female mice were treated with either DMSO (n = 4) or 0.75 mg/kg BZ (n = 4) on days 0, 3, and 6. The number of LLPCs in BM (left panel) or spleen (right panel) on day 7 (33–35 weeks of age) were studied by flow cytometry. **c**,**d**, Strong downregulation of IgGs by BZ. *Lyn*$^{-/-}$ male mice were treated as in **b** (n = 4 [DMSO] and n = 5 [BZ]). After day 7 (week 1), mice were further administered with DMSO or BZ (continued BZ) twice per week until week 5. Serum concentration of anti-dsDNA IgG (**c**) and total IgG (**d**) at the indicated time points were analyzed by ELISA. **e**, ISG expression. *Ifit1* mRNA levels in peripheral blood from the mice in Fig. 4d (n = 11 [*Lyn*$^{-/-}$*Irf5*$^{fl/fl}$] and 7 [*Lyn*$^{-/-}$*Irf5*$^{fl/fl}$*CreER*] per time point) were quantified by RT-qPCR. Horizontal bars (**b**–**e**) denote mean ± SEM. Dashed lines (**c**,**d**,**e**) indicate the mean data from WT mice. $^*P < 0.05$, $^{**}P < 0.01$ (two-sided Student's *t*-test in **b**; two-sided paired *t*-test in **c**,**d**,**e**).

Supplementary Figure 10

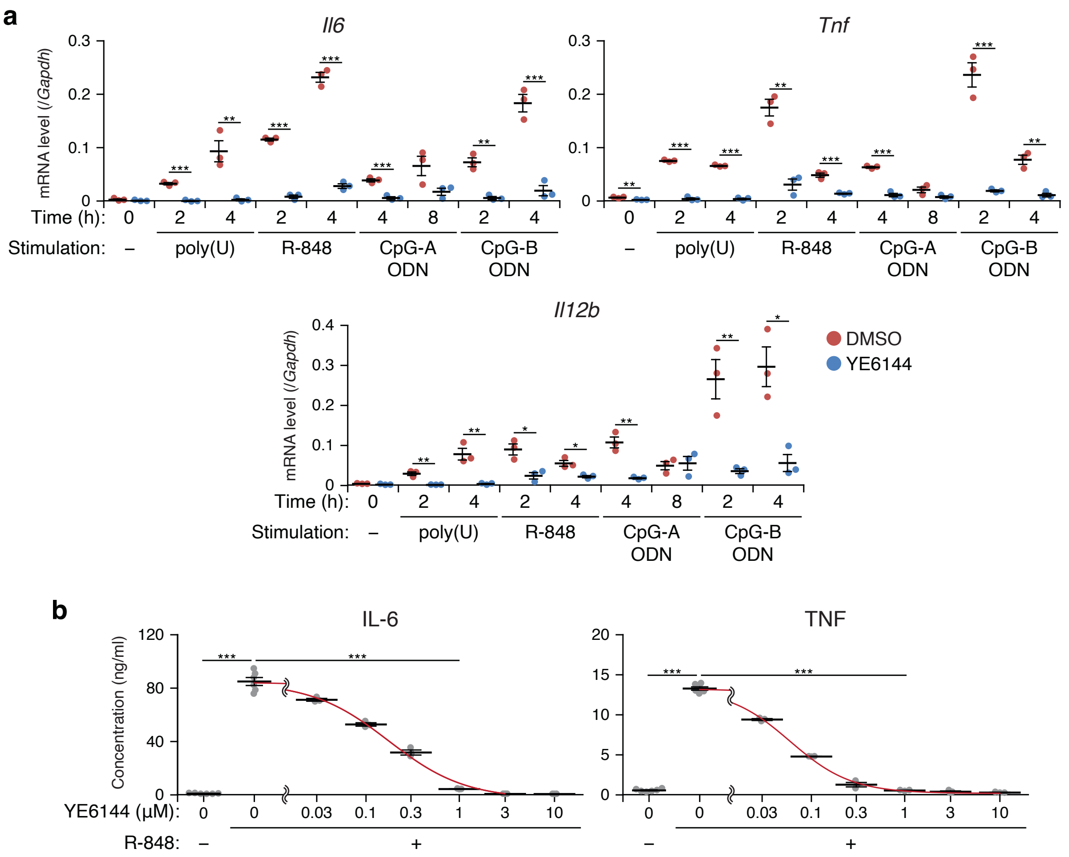

Supplementary Figure 10. The impact of the IRF5 inhibitor YE6144 on transcription factor activation and cytokine induction.

a,**b**, Proinflammatory cytokines. **a**, Mouse WT splenocytes were treated as in Fig. 5e. Total RNA was isolated, and the expression of *Il6*, *Tnf*, and *Il12b* was analyzed by RT-qPCR. **b**, Human HC PBMCs were pretreated as in Fig. 5f. IL-6 and TNF in the culture supernatants were quantitated by ELISA. The concentration (0.03–10 μM) of YE6144 was plotted on a logarithmic scale. The red line represents a four-parameter log-logistic dose-response curve. Data are representative of two independent experiments (n = 3 in **a** and n = 3 [YE6144] or 6 [DMSO] in **b** for each experiment). Horizontal bars represent mean ± SEM. $*P < 0.05$, $**P < 0.01$, $***P < 0.001$, ns: not significant (two-sided Student's *t*-test).

Supplementary Figure 11

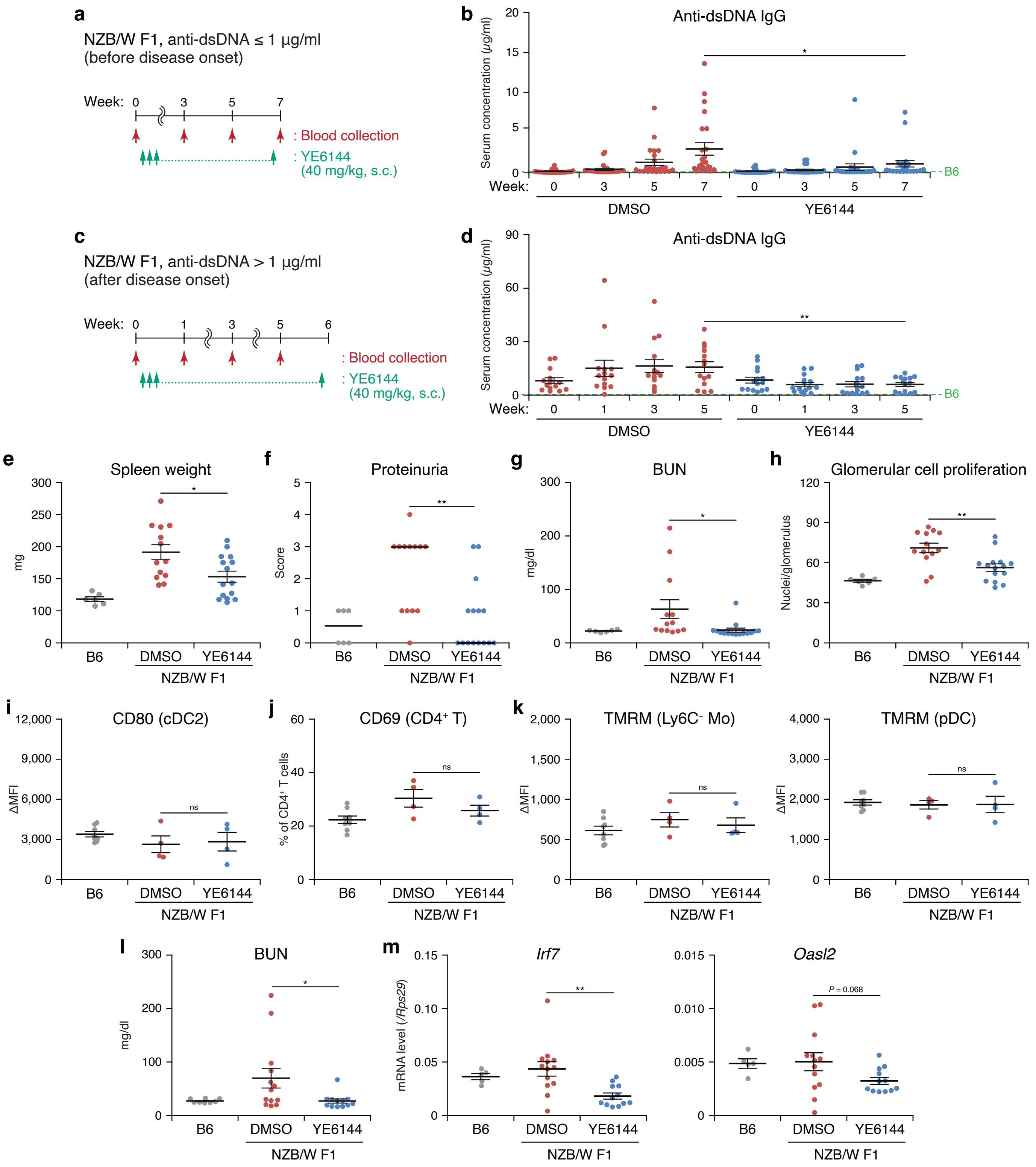

Supplementary Figure 11. The effects of YE6144 on the NZB/W F1 mouse model of SLE.
a–**k**, Effects of YE6144 alone. **a**–**d**, The scheme of YE6144 treatment alone in the NZB/W F1 mouse model and autoantibody production before (**a**,**b**) and after (**c**,**d**) the disease onset. Serum anti-dsDNA IgG levels in NZB/W F1 female mice (19 and 28 weeks of age at week 0 in a and c, respectively) treated with either DMSO (n = 23 and 14 in **b** and **d**, respectively) or YE6144 (n = 23 and 15) were analyzed by ELISA. The dashed line denotes the mean data from WT B6 mice (n = 4 and 6). **e**–**h**, Spleen weight (**e**), proteinuria (**f**), serum BUN (**g**), and kidney pathology (**h**) of mice at week 6 in **c**. **i**–**k**, CD80 expression level in cDC2s (**i**), frequency of CD69^{+}CD4^{+} T cells (**j**), and TMRM signal in the indicated cell types from the spleen (**k**) in WT B6 (n = 8) and NZB/W F1 female mice (32 weeks of age at week 0 in **c**) at 4–7 weeks after the initial DMSO (n = 4) or YE6144 (n = 4) injection (36–39 weeks of age). **l**,**m**, Effects of YE6144 in combination with BZ. Serum BUN (**l**), *Irf7*, and *Oasl2* mRNA levels in the peripheral blood (**m**) from the mice in Fig. 6h. Horizontal bars represent mean ± SEM (**b**,**d**,**e**,**g**–**m**) or median (**f**). Data in **i**–**k** were pooled from four independent experiments. *$P < 0.05$, **$P < 0.01$ (two-sided Student's *t*-test in **b**,**d**,**e**,**g**–**m**; two-sided Mann-Whitney *U* test in **f**).

Supplementary Table 1. Background information on the SLE patients and healthy controls (HC).

	SLE							HC		
	Total		AP (SLEDAI ≥ 5)		RP (SLEDAI 0–4)					
**Gender [n (%)]**								**Gender [n (%)]**		
Females	44	(76)	21	(78)	23	(74)		Females	9	(36)
Males	14	(24)	6	(22)	8	(26)		Males	16	(64)
Total	58	(100)	27	(100)	31	(100)		Total	25	(100)
**Age [range (median)]**								**Age [range (median)]**		
	9–85	(29)	9–85	(29)	10–72	(29)			20–59	(32)
**SLEDAI [range (median)]**										
	0–25	(6)	6–25	(13)	0–4	(2)				
**Anti-dsDNA [range (median)]**										
	0–1840	(19)	0–1840	(51)	0–81	(0)				
**No treatment [n (%)]**										
	11	(19)	11	(41)	0	(0)				
**Treatment [n (%)]**										
Corticosteroids										
PSL	45	(78)	15	(56)	30	(97)				
Antimalarial										
HCQ	18	(31)	7	(26)	11	(35)				
Immunosuppressants										
IVCY	16	(28)	5	(19)	11	(35)				
MMF	25	(43)	7	(26)	18	(58)				
AZP	3	(5)	1	(4)	2	(6)				
TAC	12	(21)	5	(19)	7	(23)				
Biologics										
Belimumab	2	(3)	1	(4)	1	(3)				

PSL: prednisolone, HCQ: hydroxyl chloroquine, IVCY: intravenous cyclophosphamide, MMF: mycophenolate mofetil, AZP: azathioprine, TAC: tacrolimus.

Supplementary Table 2. The list of antibodies used for nuclear-translocation analysis and flow cytometry.

Species	Fluorochrome	Antigen	Clone	Company	Dilution
Human	PE	CD141	M80	BioLegend	1:25
	PE-Cy7	HLA/DR	L243	BioLegend	1:100
	APC	CD14	M5E2	BioLegend	1:50
	APC-Cy7	CD1c	L161	BioLegend	1:50
	Brilliant Violet 421	CD123	7G3	BD Biosciences	1:100
	Brilliant Violet 605	CD303	201A	BioLegend	1:25
	Brilliant Violet 711	CD19	HIB19	BioLegend	1:50
Mouse	FITC	CD11b	M1/70	BioLegend	1:200
	FITC	PDCA-1	927	BioLegend	1:200
	PE	Control	R35-95	BD Biosciences	1:500
	PE	CD69	H1.2F3	BioLegend	1:500
	PE	CD80	16-10A1	BioLegend	1:500
	PE	TACI	8F10	BioLegend	1:500
	PE-Cy7	CD8a	53-6.7	BioLegend	1:500
	PE-Cy7	CD115	AFS98	BioLegend	1:200
	PE-Cy7	CD138	281-2	BioLegend	1:2000
	PE-Cy7	CD172a (SIRPα)	P84	BioLegend	1:500
	Alexa Fluor 647	F4/80	BM8	BioLegend	1:500
	APC	CD3ε	145-2C11	BioLegend	1:200
	APC	XCR1	ZET	BioLegend	1:500
	APC	CD45R/B220	RA3-6B2	BioLegend	1:500
	APC-Cy7	CD4	GK1.5	BioLegend	1:500
	APC-Cy7	CD11c	N418	BioLegend	1:500
	APC-Cy7	Ly6C	HK1.4	BioLegend	1:500
	Brilliant Violet 421	CD19	6D5	BioLegend	1:200
	Brilliant Violet 421	Ly6G	1A8	BioLegend	1:500
	Brilliant Violet 421	MHCII (I-A/I-E)	M5/114.15.2	BioLegend	1:2000
	Brilliant Violet 510	CD11b	M1/70	BioLegend	1:500
	Brilliant Violet 510	Ly6C	HK1.4	BioLegend	1:500

Supplementary Table 3. The list of primers for RT-qPCR.

Species	Gene	Direction	Sequence
Human	*IFI27*	Forward	5´-CCT CCA TAG CAG CCA AGA TGA TGT C-3´
		Reverse	5´-GGA TGA ACT TGG TCA ATC CGG AGA-3´
	MX1	Forward	5´-GTT GGA GGC ACT GTC AGG AGT TG-3´
		Reverse	5´-GCC TCT CCA CTT ATC TTC GTT CAC A-3´
	OAS1	Forward	5´-GAT GTG CTG CCT GCC TTT GAT G-3´
		Reverse	5´-CGA TGA GCT TGA CAT AGA TTT GGG G-3´
	GAPDH	Forward	5´-GAA ATC CCA TCA CCA TCT TCC AGG-3´
		Reverse	5´-GAG CCC CAG CCT TCT CCA TG-3´
Mouse	*Irf5*	Forward	5´-CAG TGG GTC AAC GGG GAA AAG AAA C-3´
		Reverse	5´-CTT TAG CCC AGG CCT TGA AGA TGG-3´
	Ifit1	Forward	5´-ATT CCT GCT GTT TTG GAC TCC TGT-3´
		Reverse	5´-CCA GGA CAT TAG CAA AGG GTG GAA-3´
	Irf7	Forward	5´-ACA GCA CAG GGC GTT TTA TC-3´
		Reverse	5´-GAG CCC AGC ATT TTC TCT TG-3´
	Isg15	Forward	5´-GAG CTA GAG CCT GCA GCA AT-3´
		Reverse	5´-TAA GAC CGT CCT GGA GCA CT-3´
	Oasl2	Forward	5´-CTG GAA TGT ACA GCG AGC GAG G-3´
		Reverse	5´-TTC ATC TTT CTG ATG GGG CTG TAG G-3´
	Ifna	Forward	5´-CCT GAG ARA GAA GAA ACA CAG CC-3´
		Reverse	5´-GGC TCT CCA GAY TTC TGC TCT G-3´
	Ifnb1	Forward	5´-GCT CCT GGA GCA GCT GAA TG-3´
		Reverse	5´-CGT CAT CTC CAT AGG GAT CTT GA-3´
	Il12b	Forward	5´-AGT GTG AAG CAC CAA ATT ACT C-3´
		Reverse	5´-CCC GAG AGT CAG GGG AAC T-3´
	Il6	Forward	5´-TGT TCT CTG GGA AAT CGT GGA-3´
		Reverse	5´-CTG CAA GTG CAT CAT CGT TGT-3´
	Tnf	Forward	5´-TAT GGC CCA GAC CCT CAC ACT C-3´
		Reverse	5´-CAC TTG GTG GTT TGC TAC GAC GT-3´
	Gapdh	Forward	5´-GTG TTC CTA CCC CCA ATG T-3´
		Reverse	5´-TGT CAT CAT ACT TGG CAG GTT TC-3´
	Rps29	Forward	5´-GTA CGC GAA GGA CAT AGG CTT CAT TA-3´
		Reverse	5´-AAG ACT AGC ATG ATC GGT TCC ACT T-3´
