# Supplementary material for: Genetic and chemical inhibition of IRF5 suppresses pre-existing mouse lupus-like disease
Source: Nat Commun. 2021 Jul 19;12:4379. doi: 10.1038/s41467-021-24609-4 (PMC8290003; doi:10.1038/s41467-021-24609-4)
Supplement: Supplementary file 3 — Reporting Summary [file 41467_2021_24609_MOESM3_ESM.pdf]

nature research

Corresponding author(s): Tatsuma Ban and Tomohiko Tamura

Last updated by author(s): May 26, 2021

# Reporting Summary

Nature Research wishes to improve the reproducibility of the work that we publish. This form provides structure for consistency and transparency in reporting. For further information on Nature Research policies, see our Editorial Policies and the Editorial Policy Checklist.

## Statistics

For all statistical analyses, confirm that the following items are present in the figure legend, table legend, main text, or Methods section.

| n/a | Confirmed | |
|---|---|---|
| ☐ | ☒ | The exact sample size (*n*) for each experimental group/condition, given as a discrete number and unit of measurement |
| ☐ | ☒ | A statement on whether measurements were taken from distinct samples or whether the same sample was measured repeatedly |
| ☐ | ☒ | The statistical test(s) used AND whether they are one- or two-sided<br>*Only common tests should be described solely by name; describe more complex techniques in the Methods section.* |
| ☐ | ☒ | A description of all covariates tested |
| ☐ | ☒ | A description of any assumptions or corrections, such as tests of normality and adjustment for multiple comparisons |
| ☐ | ☒ | A full description of the statistical parameters including central tendency (e.g. means) or other basic estimates (e.g. regression coefficient) AND variation (e.g. standard deviation) or associated estimates of uncertainty (e.g. confidence intervals) |
| ☐ | ☒ | For null hypothesis testing, the test statistic (e.g. *F*, *t*, *r*) with confidence intervals, effect sizes, degrees of freedom and *P* value noted<br>*Give P values as exact values whenever suitable.* |
| ☒ | ☐ | For Bayesian analysis, information on the choice of priors and Markov chain Monte Carlo settings |
| ☒ | ☐ | For hierarchical and complex designs, identification of the appropriate level for tests and full reporting of outcomes |
| ☐ | ☒ | Estimates of effect sizes (e.g. Cohen's *d*, Pearson's *r*), indicating how they were calculated |

*Our web collection on statistics for biologists contains articles on many of the points above.*

## Software and code

Policy information about availability of computer code

| | |
|---|---|
| Data collection | Flow cytometry data were acquired using FACS DIVA 8.0.1 (Aria II) or 6.1.3 (Canto II).<br>The images of fluorescent immunostaining were collected using FV-10 ASW 4.2 (Olympus).<br>The images of stained kidney sections were collected using BZ Viewer 2.52.0000.0202.0202 (Keyence).<br>Capillary-based immunoassay data acquisition was performed using Compass for SW 3.1.7 (ProteinSimple).<br>RT-qPCR data acquisition was performed using CFX Manager 3.1 (BIO-RAD).<br>Illumina Real-Time Analysis (RTA) v2 software was used for basecalling. |
| Data analysis | Flow cytometry data were analyzed using FlowJo V10.0.7 (TreeStar).<br>For nuclear translocation, fluorescent images were analyzed using CellProfiler 2.20.<br>For renal pathology, H&E, PAS, and fluorescent images were analyzed using ImageJ 1.50i (NIH).<br>RNA-seq data were converted to FASTQ file using bcl2fastq 2.20.0.422, followed by mapping the mouse genome reference sequence mm10 using STAR 2.6.0c. Raw read counts were obtained using featureCounts 1.6.2. Normalization and final read counts were obtained using edgeR 3.20.9.<br>GSEA was performed using GSEA v3.0.<br>Statistical analyses were performed in the R software version 4. |

For manuscripts utilizing custom algorithms or software that are central to the research but not yet described in published literature, software must be made available to editors and reviewers. We strongly encourage code deposition in a community repository (e.g. GitHub). See the Nature Research guidelines for submitting code & software for further information.

## Data

Policy information about availability of data

All manuscripts must include a data availability statement. This statement should provide the following information, where applicable:

- Accession codes, unique identifiers, or web links for publicly available datasets
- A list of figures that have associated raw data
- A description of any restrictions on data availability

The RNA-seq data are available in the Gene Expression Omnibus (GEO) database (accession no. GSE146334).
The public RNA-seq data of SLE patients and healthy donors were fetched from the GEO database (accession no. GSE72509).

# Field-specific reporting

Please select the one below that is the best fit for your research. If you are not sure, read the appropriate sections before making your selection.

☒ Life sciences ☐ Behavioural & social sciences ☐ Ecological, evolutionary & environmental sciences

For a reference copy of the document with all sections, see nature.com/documents/nr-reporting-summary-flat.pdf

# Life sciences study design

All studies must disclose on these points even when the disclosure is negative.

| | |
|---|---|
| Sample size | No statistical methods were used to predetermine sample size. However, the sample sizes were made as large as possible and data was routinely collected across at least two independent experiments. |
| Data exclusions | No data were excluded from analysis. |
| Replication | All experiments were repeated independently as indicated in the figure legends and the reproducibilities were confirmed. |
| Randomization | In the experiments in Fig. 1 and Supplementary Figs. 1–4, samples from SLE patients were allocated into AP-SLE and RP-SLE groups according to the SLEDAI-2K score. In the experiments in Figs. 2–4 and Supplementary Figs. 5–9, comparisons were done across mice of different genotypes with matched age and sex. In the experiments in Fig. 5 and Supplementary Fig. 10, cells were prepared from the same biological samples from humans or mice and then assigned to the experimental groups. In the experiments in Fig. 6 and Supplementary Fig. 11, the anti-dsDNA antibody concentration in NZB/W F1 mice was measured to determine the disease onset, and then mice were assigned to experimental groups so that the distribution of initial anti-dsDNA concentrations were similar. |
| Blinding | In the experiments using SLE patient specimen, the investigators (G.R.S and N.T) were blind to the clinical record until the experiments (nuclear translocation analysis and gene expression analysis) were completed. In the other experiments, blinding was not necessary as cells or mice were grouped by genotype and/or condition rather than objective allocation. |

# Reporting for specific materials, systems and methods

We require information from authors about some types of materials, experimental systems and methods used in many studies. Here, indicate whether each material, system or method listed is relevant to your study. If you are not sure if a list item applies to your research, read the appropriate section before selecting a response.

## Materials & experimental systems

| n/a | Involved in the study | |
|---|---|---|
| ☐ | ☒ | Antibodies |
| ☐ | ☒ | Eukaryotic cell lines |
| ☒ | ☐ | Palaeontology and archaeology |
| ☐ | ☒ | Animals and other organisms |
| ☐ | ☒ | Human research participants |
| ☒ | ☐ | Clinical data |
| ☒ | ☐ | Dual use research of concern |

## Methods

| n/a | Involved in the study | |
|---|---|---|
| ☒ | ☐ | ChIP-seq |
| ☐ | ☒ | Flow cytometry |
| ☒ | ☐ | MRI-based neuroimaging |

## Antibodies

| | |
|---|---|
| Antibodies used | For capillary-based immunoassay, anti-phospho IRF5 (newly generated, working concentration: 25 μg/ml), anti-IRF5 (Abcam; cat. no. ab181553, clone no. EPR17067, lot no. GR3248905-3, working concentration: 25 μg/ml), anti-IKKβ antibody (CST; cat. no. 2678S, 4, working concentration: 1.9 μg/mL), anti-GAPDH (Abcam; ab8245, cat. no. 6C5, GR3207992-2, working concentration: 10 μg/ml), HRP-conjugated anti-Rabbit IgG (ProteinSimple; cat. no. DM-001, lot no. 10889, no dilution), and HRP-conjugated anti-Mouse IgG (GE Healthcare; cat. no. NA931-1ML, dilution 1:50) were used. |

For nuclear translocation analysis, anti-IRF5 (Abcam; ab21689, lot no. GR126979-3, working concentration: 3 μg/ml), anti-p65 (Santa Cruz; cat. no. sc-372X, lot no. H0714, working concentration: 3 μg/ml), normal rabbit IgG (CST; cat. no. 2729, 6, working concentration: 3 μg/ml), and AF488-conjugated anti-rabbit F(ab')2 fragment (Jackson Immuno Research; cat. no. 711-546-152, 133588, working concentration: 3 μg/ml) were used.
For anti-dsDNA IgG ELISA, mouse anti-dsDNA IgG (Santa Cruz; cat. no. sc-58749, HYB331-01, lot no. K2717, standard concentration: 100, 50, 25, 12.5, 6.3, 3.1, 1.6, 0.8 ng/ml), and HRP-conjugated anti-Mouse IgG-Fc antibody (Bethyl; cat. no. A90-131P, lot no. 39, dilution 1:20000) were used.
Antibodies against cell surface antigen were listed below.
The following antibodies were from BioLegend:
human CD141 (clone M80, catalog numbers 344104, dilution 1:25),
human HLA/DR (clone L243, catalog numbers 307616, dilution 1:100),
human CD14 (clone M5E2, catalog numbers 301808, dilution 1:50),
human CD1c (clone L161, catalog numbers 331520, dilution 1:50),
human CD303 (clone 201A, catalog numbers 354224, dilution 1:25),
human CD19 (clone HIB19, catalog numbers 302246, dilution 1:50),
mouse CD11b (clone M1/70, catalog numbers 101206, dilution 1:200),
mouse PDCA-1 (clone 927, catalog numbers 127008, dilution 1:200),
mouse CD69 (clone H1.2F3, catalog numbers 104508, dilution 1:500),
mouse CD80 (clone 16-10A1, catalog numbers 104708, dilution 1:500),
mouse TACI (clone 8F10, catalog numbers 133404, dilution 1:500),
mouse CD8a (clone 53-6.7, catalog numbers 100722, dilution 1:500),
mouse CD115 (clone AFS98, catalog numbers 135524, dilution 1:200),
mouse CD138 (clone 281-2, catalog numbers 142504, dilution 1:2000),
mouse CD172a (clone P84, catalog numbers 144012, dilution 1:500),
mouse F4/80 (clone BM8, catalog numbers 123122, dilution 1:500),
mouse CD3ε (clone 145-2C11, catalog numbers 100312, dilution 1:200),
mouse XCR1 (clone ZET, catalog numbers 100330, dilution 1:500),
mouse CD45R/B220 (clone RA3-6B2, catalog numbers 103212, dilution 1:500),
mouse CD4 (clone GK1.5, catalog numbers 100414, dilution 1:500),
mouse CD11c (clone N418, catalog numbers 117318, dilution 1:500),
mouse Ly6C (clone HK1.4, catalog numbers 128026, dilution 1:500),
mouse CD19 (clone 6D5, catalog numbers 115549, dilution 1:200),
mouse Ly6G (clone 1A8, catalog numbers 127628, dilution 1:500),
mouse MHCII (I-A/I-E) (clone M5/114.15.2, catalog numbers 107619, dilution 1:2000),
mouse CD11b (clone M1/70, catalog numbers 101263, dilution 1:500),
mouse Ly6C (clone HK1.4, catalog numbers 128033, dilution 1:500)
The following antibodies were from BD Biosciences:
human CD123 (clone 7G3, catalog numbers 563362, dilution 1:100),
Control IgG (clone M1/70, catalog numbers 557397, dilution 1:500)

Validation

All commercially available antibodies were validated by the manufacturer.
anti-IRF5 (Abcam; ab181553, clone EPR17067) [https://www.abcam.co.jp/irf5-antibody-epr17067-ab181553.html]
anti-IKKβ antibody (CST; 2678S) [https://www.cellsignal.jp/products/primary-antibodies/ikkb-l570-antibody/2678]
anti-GAPDH (Abcam; ab8245, clone 6C5) [https://www.abcam.co.jp/gapdh-antibody-6c5-loading-control-ab8245.html]
HRP-conjugated anti-Rabbit IgG (ProteinSimple; DM-001) [https://shop.proteinsimple.com/anti-rabbit-detection-module-for-wes-peggy-sue-or-sally-sue.html]
HRP-conjugated anti-Mouse IgG (GE Healthcare; NA931-1ML) [https://www.cytivalifesciences.com/en/us/shop/protein-analysis/blotting-and-detection/blotting-standards-and-reagents/amersham-ecl-hrp-conjugated-antibodies-p-06260]
anti-IRF5 (Abcam; ab21689) [https://www.abcam.co.jp/irf5-antibody-ab21689.html]
anti-p65 (Santa Cruz; sc-372X) [https://datasheets.scbt.com/sc-372.pdf]
normal rabbit IgG (CST; 2729) [https://www.cellsignal.jp//products/primary-antibodies/normal-rabbit-igg/2729]
AF488-conjugated anti-rabbit F(ab')2 fragment (Jackson Immuno Research; 711-546-152) [https://www.jacksonimmuno.com/catalog/products/711-546-152]
mouse anti-dsDNA IgG (Santa Cruz; sc-58749, clone HYB331-01) [https://datasheets.scbt.com/sc-58749.pdf]
HRP-conjugated anti-Mouse IgG-Fc antibody (Bethyl; A90-131P) [https://www.bethyl.com/product/A90-131P/Mouse+IgG-Fc+Fragment+Antibody_Seconary+Antibody]
anti-human CD141 (BioLegend, clone M80, 344104) [https://www.biolegend.com/ja-jp/products/pe-anti-human-cd141-thrombomodulin-antibody-6107]
anti-human HLA/DR (BioLegend, clone L243, 307616) [https://www.biolegend.com/ja-jp/products/pe-cyanine7-anti-human-hla-dr-antibody-2862]
anti-human CD14 (BioLegend, clone M5E2, 301808) [https://www.biolegend.com/ja-jp/products/apc-anti-human-cd14-antibody-793]
anti-human CD1c (BioLegend, clone L161, 331520) [https://www.biolegend.com/ja-jp/products/apc-cyanine7-anti-human-cd1c-antibody-6937]
anti-human CD303 (BioLegend, clone 201A, 354224) [https://www.biolegend.com/ja-jp/products/brilliant-violet-605-anti-human-cd303-bdca-2-antibody-13062]
anti-human CD19 (BioLegend, clone HIB19, 302246) [https://www.biolegend.com/ja-jp/products/brilliant-violet-711-anti-human-cd19-antibody-8519]
anti-mouse CD11b (BioLegend, clone M1/70, 101206) [https://www.biolegend.com/ja-jp/products/fitc-anti-mouse-human-cd11b-

antibody-347]
anti-mouse PDCA-1 (BioLegend, clone 927, 127008) [https://www.biolegend.com/ja-jp/products/fitc-anti-mouse-cd317-bst2-pdca-1-antibody-6349]
anti-mouse CD69 (BioLegend, clone H1.2F3, 104508) [https://www.biolegend.com/ja-jp/products/pe-anti-mouse-cd69-antibody-265]
anti-mouse CD80 (BioLegend, clone 16-10A1, 104708) [https://www.biolegend.com/ja-jp/products/pe-anti-mouse-cd80-antibody-43]
anti-mouse TACI (BioLegend, clone 8F10, 133404) [https://www.biolegend.com/ja-jp/products/pe-anti-mouse-cd267-taci-antibody-5807]
anti-mouse CD8a (BioLegend, clone 53-6.7, 100722) [https://www.biolegend.com/ja-jp/products/pe-cyanine7-anti-mouse-cd8a-antibody-1906]
anti-mouse CD115 (BioLegend, clone AFS98, 135524) [https://www.biolegend.com/ja-jp/products/pe-cyanine7-anti-mouse-cd115-csf-1r-antibody-12376]
anti-mouse CD138 (BioLegend, clone 281-2, 142504) [https://www.biolegend.com/ja-jp/products/pe-anti-mouse-cd138-syndecan-1-antibody-7519]
anti-mouse CD172a (BioLegend, clone P84, 144012) [https://www.biolegend.com/ja-jp/products/pe-anti-mouse-cd172a-sirpalpha-antibody-9801]
anti-mouse F4/80 (BioLegend, clone BM8, 123122) [https://www.biolegend.com/ja-jp/products/alexa-fluor-647-anti-mouse-f4-80-antibody-4074]
anti-mouse CD3ε (BioLegend, clone 145-2C11, 100312) [https://www.biolegend.com/ja-jp/products/apc-anti-mouse-cd3epsilon-antibody-21]
anti-mouse XCR1 (BioLegend, clone ZET, 100330) [https://www.biolegend.com/ja-jp/products/apc-cyanine7-anti-mouse-cd3epsilon-antibody-6070]
anti-mouse CD45R/B220 (BioLegend, clone RA3-6B2, 103212) [https://www.biolegend.com/ja-jp/products/apc-anti-mouse-human-cd45r-b220-antibody-442]
anti-mouse CD4 (BioLegend, clone GK1.5, 100414) [https://www.biolegend.com/ja-jp/products/apc-cyanine7-anti-mouse-cd4-antibody-1964]
anti-mouse CD11c (BioLegend, clone N418, 117318) [https://www.biolegend.com/ja-jp/products/pe-cyanine7-anti-mouse-cd11c-antibody-3086]
anti-mouse Ly6C (BioLegend, clone HK1.4, 128026) [https://www.biolegend.com/ja-jp/products/apc-cyanine7-anti-mouse-ly-6c-antibody-6758]
anti-mouse CD19 (BioLegend, clone 6D5, 115549) [https://www.biolegend.com/ja-jp/products/brilliant-violet-421-anti-mouse-cd19-antibody-7160]
anti-mouse Ly6G (BioLegend, clone 1A8, 127628) [https://www.biolegend.com/ja-jp/products/brilliant-violet-421-anti-mouse-ly-6g-antibody-7161]
anti-mouse MHCII (I-A/I-E) (BioLegend, clone M5/114.15.2, 107619) [https://www.biolegend.com/ja-jp/products/pacific-blue-anti-mouse-i-a-i-e-antibody-3136]
anti-mouse CD11b (BioLegend, clone M1/70, 101263) [https://www.biolegend.com/ja-jp/products/brilliant-violet-510-anti-mouse-human-cd11b-antibody-7993]
anti-mouse Ly6C (BioLegend, clone, 128033) [https://www.biolegend.com/ja-jp/products/brilliant-violet-510-anti-mouse-ly-6c-antibody-8726]
anti-human CD123 (BD Biosciences, clone 7G3, 563362) [https://www.bdbiosciences.com/jp/applications/research/b-cell-research/surface-markers/human/bv421-mouse-anti-human-cd123-7g3/p/563362]
Rat control IgG (BD Biosciences, clone M1/70, 557397) [https://www.bdbiosciences.com/us/applications/research/stem-cell-research/mesenchymal-stem-cell-markers-bone-marrow/mouse/negative-markers/pe-rat-anti-cd11b-m170/p/557397]
To confirm the specificity of our newly generated monoclonal antibody against phospho-IRF5, we showed that the IKKβ-induced IRF5 phosphorylation detected by the antibody disappeared when the Serine 446 of IRF5 was substituted by alanine (Extended Data Figure 1).

## Eukaryotic cell lines

Policy information about cell lines

| | |
|---|---|
| Cell line source(s) | HEK293T cells used in this study were obtained from Dr. Mutsuhiko Minami's laboratory, Yokohama City University Graduate School of Medicine. Commercial source: Riken BRC, RCB2202. |
| Authentication | Authentication was not performed. |
| Mycoplasma contamination | Not tested |
| Commonly misidentified lines (See ICLAC register) | No commonly misidentified cell lines were used. |

## Animals and other organisms

Policy information about studies involving animals; ARRIVE guidelines recommended for reporting animal research

| | |
|---|---|
| Laboratory animals | WT, Lyn−/− , Lyn−/− Irf5+/−, Lyn−/− Irf5−/−, Lyn−/− Ifnar1+/−, Lyn−/− Ifnar1−/−, Lyn−/− Irf5fl/fl, and Lyn−/− Irf5fl/fl CreER mice (male and female, C57BL/6 background), and NZB/W F1 mice (female) were used at 11 to 51 weeks of age. Mice were housed in a 12h/12h light/dark cycle at 24.0 +/- 1.0 degrees Celsius and a humidity range of 50.0 +/- 5.0%. |
| Wild animals | Wild animals were not analyzed in this study. |

| | |
|---|---|
| Field-collected samples | Field-collected samples were not analyzed in this study. |
| Ethics oversight | All animal experiments were performed in accordance with the Guidelines for Proper Conduct of Animal Experiments (Science Council of Japan), and all protocols were approved by the institutional review boards of Yokohama City University (protocols F-A-17-018 and F-A-20-043). |

Note that full information on the approval of the study protocol must also be provided in the manuscript.

## Human research participants

Policy information about studies involving human research participants

| | |
|---|---|
| Population characteristics | Asian pediatric or adult patients with SLE, or adult healty donors participated in this study. Gender, age and clinical record were described in Supplementary Table 1. |
| Recruitment | SLE patients who fulfilled at least four of the ACR 1997 revised classification criteria at Yokohama City University Hospital were recruited. Informed consent was obtained from all subjects or their guardians. There was no self-selection bias. |
| Ethics oversight | The research on human subjects conducted in this study was approved by the Yokohama City University Certified Institutional Review Board (approval no. A120322001). |

Note that full information on the approval of the study protocol must also be provided in the manuscript.

## Flow Cytometry

### Plots

Confirm that:

- [x] The axis labels state the marker and fluorochrome used (e.g. CD4-FITC).
- [x] The axis scales are clearly visible. Include numbers along axes only for bottom left plot of group (a 'group' is an analysis of identical markers).
- [x] All plots are contour plots with outliers or pseudocolor plots.
- [x] A numerical value for number of cells or percentage (with statistics) is provided.

### Methodology

| | |
|---|---|
| Sample preparation | Preparation of human PBMCs, mouse splenocytes, and BM cells was described in Methods. |
| Instrument | Human Mo, pDCs, cDCs, and B cells were sorted from PBMCs using FACSAria II Cell Sorter (BD). Mouse splenic cDC1s, cDC2s, pDCs, CD4+ T cells, CD8+ T cells, B cells, Ly6C–Mo, Ly6C+Mo, granulocytes, macrophages and LLPCs, and BM LLPCs were analyzed using FACSCanto II Flow Cytometer (BD). |
| Software | Flow cytometry data were acquired using FACS DIVA 8.0.1 (Aria II) or 6.1.3 (Canto II). Further analysis was performed using FlowJo V10.0.7 (TreeStar). |
| Cell population abundance | The purity of the post-sort fractions determined by flow cytometry was more than 99%. |
| Gating strategy | Gating strategy was described in Supplementary Figs 1a, 5a, 5b, 7a, and 9a, and Methods. |

- [x] Tick this box to confirm that a figure exemplifying the gating strategy is provided in the Supplementary Information.
